# Impact of Dam Lactation Number on Colostrum Quality, Calf Growth, and Economic Performance in Holstein Cows

**DOI:** 10.3390/vetsci13060600

**Published:** 2026-06-20

**Authors:** Andrea García-Mendoza, Milagros González-Hernández, Delia X. Vega-Manriquez, Erika Félix-Santiago, María del Refugio Pérez-Barba, César A. Rosales-Nieto

**Affiliations:** 1 Facultad de Agronomía y Veterinaria, Universidad Autónoma de San Luis Potosí, San Luis Potosí 78321, Mexico; 2 Facultad de Ciencias Químicas, Universidad Autónoma de San Luis Potosí, San Luis Potosí 78210, Mexico

**Keywords:** colostrum, number of births, coccidiosis, development at weaning

## Abstract

This study investigated the associations among dam lactation number, passive immunity transfer, internal parasite burden, and weaning performance in female calves. Early consumption of high-quality colostrum is critical for effective passive immunity transfer, calf health, and long-term productivity. Colostrum composition may vary with management practices and dam lactation number, potentially influencing immunity and disease susceptibility. Forty-five cows and their female offspring (n = 45) were allocated to three groups by lactation number: first lactation (LAC1), second lactation (LAC2), and third lactation (LAC3) (15 cows and 15 calves per group). Calf birth weight was recorded. Colostrum immunoglobulin G (IgG) concentration was measured using a colostrometer and refractometer, and chemical composition (fat, protein, and non-fat solids) was analyzed. Fecal samples collected at 30, 60, and 90 days of age were examined for *Eimeria* spp. Colostrum IgG concentration, °Brix percentage, specific gravity, and non-fat solids did not differ among lactation groups (*p* > 0.05). LAC3 cows had higher colostrum protein content, while LAC1 cows had greater fat concentration (*p* < 0.05). Calves from LAC1 dams were lighter at birth than those from LAC2 and LAC3 dams (*p* < 0.001). The prevalence of *Eimeria* spp. was not influenced by lactation number, birth weight, or colostrum quality (*p* > 0.05), but treatment costs were higher in calves from LAC3 dams (*p* < 0.01). In conclusion, lactation number affected colostrum composition and calf growth but did not alter IgG concentration, underscoring the importance of effective colostrum management to improve calf performance and dairy system sustainability.

## Simple Summary

This study investigated the influence of lactation number (first, second, or third) on colostrum quality, calf immunity, parasite load, and growth in Holstein calves. Colostrum intake immediately after birth is critical, as it supplies antibodies that protect calves from disease and promote healthy development. The composition of colostrum may vary according to the cow’s lactation number and management practices. Forty-five cows and their female offspring (n = 45) were assigned equally to groups based on the number of lactations. Data were collected on calf birth weight, colostrum quality (including immunoglobulin G [IgG], fat, protein, and solids), and the presence of *Eimeria* spp. at multiple stages up to 90 days. The findings indicated that IgG concentrations, representing passive immunity transfer, did not differ among lactation groups. In contrast, colostrum composition varied: third-lactation cows produced colostrum with higher protein content, while first-lactation cows had higher fat levels. Calves born to first-lactation cows were lighter and smaller at birth than those from older cows. *Eimeria* spp. prevalence was not influenced by lactation number, calf weight, or colostrum quality. However, treatment costs were greater for calves from third-lactation cows. In summary, lactation number affected colostrum nutrient composition and calf growth but did not impact immunity transfer, underscoring the significance of effective colostrum management.

## 1. Introduction

Neonatal survival, health, and lifetime productivity in dairy cattle are strongly influenced by events occurring during the perinatal period, among which colostrum intake is critical [1,2]. Due to the epitheliochorial nature of the bovine placenta, transplacental transfer of immunoglobulins does not occur, and calves are born agammaglobulinemic [3]. This makes calves depend entirely on the timely ingestion and intestinal absorption of colostral immunoglobulin G (IgG) to acquire passive immunity [4]. Failure of adequate passive transfer remains a major risk factor for neonatal morbidity, impaired growth, and increased mortality [5]. This continues to represent a significant challenge in both dairy and beef production systems worldwide [6].

Colostrum is a complex biological fluid containing immunoglobulins, nutrients, hormones, growth factors, and other bioactive compounds that collectively support gastrointestinal maturation, metabolic adaptation, and early postnatal development [7,8]. Lactation number is an important maternal factor influencing colostrum composition. Multiparous cows are generally assumed to produce higher-quality colostrum due to cumulative antigen exposure and greater mammary secretory capacity [9,10,11,12,13]. In contrast, primiparous cows may be limited by incomplete mammary development and ongoing somatic growth [14,15]. However, few studies have simultaneously evaluated the immunological quality of colostrum, its chemical composition, calf growth, health outcomes, and associated economic implications within a single experimental framework.

Early-life growth performance and reproductive efficiency are increasingly recognized as indicators of prenatal conditions and early postnatal nutrition [16,17,18]. Traits such as birth weight, preweaning growth rate, and skeletal development (e.g., height at weaning) are closely associated with placental efficiency, colostrum intake, and milk nutrient supply, with long-term consequences for survivability, age at first calving, and lifetime productivity [1,16,19,20]. These outcomes may respond differently to the immunological and nutritional components of colostrum, highlighting the need to better delineate their respective contributions. Neonatal susceptibility to enteric pathogens remains a substantial economic burden in dairy systems, particularly when passive immunity transfer is inadequate [21,22]. Diarrhea accounts for more than half of morbidity and nearly one-third of mortality in preweaned dairy calves, with lasting negative effects on growth and productivity [23]. Although colostrum-derived antibodies are critical for protection against many extracellular pathogens, their role in modulating coccidial infections caused by intracellular protozoa such as *Eimeria* spp. is less well understood. It remains unclear whether variation in colostrum quality associated with dam lactation number influences the prevalence or severity of such infections. These issues are particularly relevant in countries such as Mexico and somewhere else, where dairy production is economically important but insufficient to meet domestic demand. Despite advances in genetics, nutrition, and herd management, increased milk yield has often been accompanied by reduced reproductive efficiency, prompting the adoption of strategies to accelerate replacement heifer development and reduce age at first calving [18,24,25]. In this context, optimizing early-life nutrition and immunity through effective colostrum management is essential, as inadequate passive transfer predisposes calves to disease, increases treatment costs, and compromises the efficiency of replacement programs.

Previous work by our group demonstrated lower colostrum quality and reduced efficiency of passive immune transfer in calves born to primiparous cows compared with those born to multiparous cows [10]. However, postnatal growth to weaning was not consistently affected by maternal parity, suggesting that factors beyond IgG concentration contribute to calf development. Therefore, it remains unclear whether dam lactation number directly influences immune transfer, parasitic burden, postnatal growth trajectories, and associated economic outcomes in dairy calves. Accordingly, the objective of this study was to evaluate the effect of dam lactation number on colostrum IgG concentration, assessed using field-applicable methods (colostrometer and refractometer) [10,26], colostrum chemical composition, calf birth weight, preweaning growth performance, and the prevalence of *Eimeria* spp. infection during the preweaning period, as well as related treatment costs. We hypothesized that, under standardized management conditions, colostrum IgG concentration would not differ substantially among lactation groups. Additionally, differences in the nutritional composition of colostrum would be associated with variation in growth performance, health outcomes, and economic returns in dairy calves.

## 2. Materials and Methods

### 2.1. Ethical Approval

All experimental procedures adhered to the Technical Specifications for the Production, Care, and Use of Laboratory Animals of the Official Mexican Standards [27]. The Institutional Animal Care and Use Committee from the University of San Luis Potosí approved the protocol (CICUAE/2023/02; approved 23 October 2023).

### 2.2. General Study Conditions

The study was conducted on a commercial dairy farm in Central Mexico (21°73′ N, 100°96′ W). The climate in this location is semi-arid and temperate, with a defined winter season. The region has an average annual temperature of 16.7 °C and annual precipitation of approximately 361 mm. Relative humidity ranges from 45% in April to 90% during the rainy season. The farm has 3000 Holsteins and Jerseys, and around 2000 are on the production line.

### 2.3. Animals and Treatments

A total of 45 Holstein cows and their female calves (n = 45) were selected based on the number of parturitions or lactations. Productive and reproductive records maintained by the farm enabled the formation of three treatment groups: 15 primiparous cows in their first lactation (LAC1), 15 multiparous cows in their second lactation (LAC2), and 15 multiparous cows in their third lactation (LAC3). All cows calved during November 2023. This allowed us to eliminate the confounding effect of season on colostrum composition [13]. Primiparous cows from LAC1 were bred for the first time at 14 months of age, resulting in calving at approximately 23 months. Cows from LAC2 had a mean age of thirty-five months and were classified as healthy. Cows from LAC3 had a mean age of forty-seven months and were also classified as healthy. Primiparous cows were inseminated with Holstein calving ease semen, while multiparous cows received Holstein semen. Cows nearing calving were allowed to calve in the dry cow pen without human intervention. At birth, contact between the mother and calf was permitted for a period of 10 min to encourage mother-calf bonding. At birth, the calf’s weight, delivery type, and sex were recorded.

### 2.4. Management of Primiparous and Multiparous Cows at Calving

Cows nearing calving were monitored at random intervals by technicians to observe signs of impending parturition, such as restlessness, isolation, tail raising, contractions, and fetal membrane or leg observations, without intervening in the calving process.

Primiparous cows were managed in 30 × 30 m^2^ pens with 85% dirt surface and 15% concrete surface. The diet meets the nutritional requirements for both fetal development and maintenance [28]. Thirty days before parturition, pregnant cows were moved to a smaller pen, where they were offered a diet richer in protein and energy, providing 2.2 Mcal of metabolizable energy and 14.1% crude protein (Table 1). The diet’s ingredients were mixed using a feed mixer wagon and delivered along a concrete feed bunk within the pen. Primiparous cows had ad libitum access to both feed and water, and feed availability was maintained continuously.

Multiparous cows went through a 40 d dry period before parturition. The diet provided during the dry period satisfied protein and energy maintenance requirements (60% sorghum stubble and 40% corn silage [28]). Two weeks before parturition, the diet was modified to increase protein content and starch levels. The diet contributes 2.2 Mcal of metabolizable energy and 14.1% of crude protein (Table 1). Multiparous cows had ad libitum access to both feed and water. Feed availability was maintained continuously.

All primiparous and multiparous cows in the facility were vaccinated twice a year against bovine viral rhinotracheitis, bovine viral diarrhea BVD strains 1 and 2, bovine respiratory syncytial virus (BRSV), clostridiosis, and pneumonic pasteurellosis. Deworming was not applied. Pregnant cows calved in their pen. Cows in labor were not assisted unless dystocic parturition was detected. Once parturition occurred, the dam and calf were in contact for 10 min (see below).

### 2.5. Calf Management from Birth to Weaning

After birth, in accordance with standard practice, direct contact was allowed for 10 min to facilitate a mother-calf bond [29]. After this, the calves were removed, weighed on a digital scale, and placed in individual hutches. Hutches had been disinfected with bleach and a commercial disinfectant (QumicaVita^®^; Queretaro, Mexico) in advance, and the calves were kept there until weaning. The hutches were spaced about 1.5 mt apart. Immediately after birth, the umbilical cords of the calves were disinfected with a 10% iodine solution. Calves received their initial colostrum feeding within 2 h of birth, consisting of previously collected colostrum from their mothers and administered at 10% of their birth body weight. Subsequent colostrum feedings were provided at 24, 36, and 48 h postpartum at the same rate. Trained personnel administered colostrum using a feeding bottle. Following colostrum administration, calves were fed pasteurized milk twice daily (approximately 2 L per feeding) until weaning. In addition to milk, calves had ad libitum access to water and a commercial calf starter concentrate (Vimifos^®^ [Jalisco, Mexico], Vimicalf line; 88% dry matter, 20% crude protein, 3.5% crude fat, 6.5% ash, 0.8% calcium, and 0.7% phosphorus). The starter concentrate was initially offered at 0.5 kg per day and gradually increased to 2.0 kg per day as calves matured. The concentrate was mixed with alfalfa hay, which was provided in limited quantities to facilitate the gradual introduction of forage and promote rumen development. Weaning was performed individually at 90 days of age to allow females to reach a desirable height. All calves were administered NASALGEN IP nasal suspension (Merk & Co; Rahway, NJ, USA), a vaccine against infectious bovine rhinotracheitis and bovine parainfluenza type 3, with 2 mL administered intranasally in one nostril, and BOVILIS RB-51 (Merk & Co; Rahway, NJ, USA), a vaccine containing the live, lyophilized RB-51 strain of Brucella abortus. The vaccine was applied when the calves were 1 week old. The calves were weighed again at weaning (90 days post-birth) to determine weight gain during the pre-weaning period.

### 2.6. Colostrum Quality and Composition

Within the first hour after calving, all cows and calves were separated, the dam was milked, and colostrum was collected to feed the newborn calf. From the same colostrum, a subsample was separated and used to determine quality and composition (see below). Data on colostrum yield were not recorded because the number of lactations influences it [13,30].

### 2.7. Calorimetry

After colostrum was milked, a 250 mL sample was collected and placed in a KRUUSE^®^ (Langeskov, Denmark) colostrum densimeter. The densimeter was then inserted into the sample to obtain a reading of the colostrum quality. The interpretation of the reading was as follows: colostrum with a specific gravity (SG) less than 1035 is considered low-quality (red zone), an SG between 1035 and 1045 corresponds to medium-quality colostrum (green zone), and an SG greater than 1045 corresponds to good-quality colostrum. This data was recorded for later determination of the amount of immunoglobulins present [10,26].

### 2.8. Refractometry

After the colostrum was milked, a 2 mL sample was taken and placed in the portable refractometer (ATC 0–50% °Brix). The reading was taken and recorded for later determination of the amount of immunoglobulins present [10,26,31].

### 2.9. Colostrum Evaluation

The chemical composition of the colostrum was evaluated through chemical analysis (Ecomilk^®^, Milkana KAM 98-2A, Satara, Maharashtra, India) at the laboratory of the Faculty of Chemical Sciences at the University of San Luis Potosi. A 15% colostrum dilution in distilled water was prepared, and the percentages of fat, non-fat solids, protein, and density were determined.

### 2.10. Parasitic Load

Three fecal samples were taken during the pre-weaning period from the calves at days 30, 60, and 90 (weaning). These samples were stored under refrigeration (3 °C) for subsequent analysis to determine the parasitic load during this period. The determination of parasitic load was performed using the McMaster technique, which involves counting parasite oocysts; it is a quantitative test that yields the number of eggs per gram of feces. This technique is based on the principle of flotation, where the eggs present in each stool sample, when exposed to a supersaturated sodium chloride solution, separate from the fecal mass and rise to the surface of the liquid [32]. This test was conducted at the Parasitology Laboratory of the Faculty of Agronomy and Veterinary Medicine at the Autonomous University of San Luis Potosí.

### 2.11. Economic Investment

The calves were monitored to assess their health status, and records were kept of the treatments applied during the pre-weaning period to determine the economic investment. The most common condition was diarrhea.

### 2.12. Statistical Analysis

Data were analyzed using the SAS statistical package, SAS version 9.3 [33]. Each cow and calf were considered an experimental unit. Data normality was assessed using the Shapiro–Wilk test (PROC UNIVARIATE). Colostrum quality (Gravity, density, IgG content [Colostrometer and Refractometer]) and composition (Protein, fat, NFS, and density), birth weight, weight gain to weaning, weaning weight, weaning height, *Eimeria* spp. prevalence (at day 30, 60, and 90) and economic investment as dependent variables were analyzed using linear mixed model procedures (PROC MIXED). The fixed effects in the model were treatment (1st, 2nd, or 3rd lactation). Colostrum quality and composition and birth weight were included as covariates in the model for weight-to-weaning, weaning weight, high, and economic investment. Differences among treatments were analyzed using PROC GLM.

The correlations among variables were predicted using PROC GLM with the MANOVA option (Pearson) to remove major fixed effects. It was considered strong if the correlation value was >0.5 and close to 1.0. It was considered low when the correlation value was <0.5, and weak when it approached 0.0. The parity was considered a fixed effect. All 2-way interactions among the fixed effects were included in each model, and non-significant (*p* > 0.05) interactions were removed from the model.

## 3. Results

### 3.1. Colostrum Immunoglobulin Content

IgG concentration measured by colostometer and refractometer, °Brix percentage in colostrum, and colostrum gravity did not differ among dam lactation numbers (*p* > 0.05; Table 2).

### 3.2. Chemical Composition of Colostrum

Colostrum from LAC3 cows had a higher protein content than that of cows from LAC1 or LAC2 (*p* < 0.05; Table 3). Conversely, colostrum from LAC1 had a higher fat content (*p* < 0.05; Table 3) than that of LAC2 and LAC3. No differences were found in lactation numbers regarding colostrum density and non-fat solids (NFS) content (*p* > 0.05; Table 3).

### 3.3. Offspring Weight Variables

We observed highly significant differences in dam lactation number for birth weight (*p* < 0.001), weight gain at weaning (*p* < 0.01), weaning weight (*p* < 0.001), and height at weaning (*p* < 0.01; Table 4). In all the variables assessed, calves from LAC1 dams were lighter and shorter than those from dams in LAC2 and LAC3 (Table 4).

Weight gain to weaning and weaning weight were not influenced by birth weight or colostrum quality (Gravity, density, IgG content [Colostrometer and Refractometer]) and composition (Protein, fat, NFS, and density; *p* > 0.05). Skeletal development (height) was positively influenced by the concentration of IgG (Colostrometer reading; *p* < 0.01), body weight gains to weaning (*p* < 0.01), and weaning weight (*p* < 0.001). The height at weaning increased by 1.5 cm for each 20 mg/mL increase in IgG concentration. The height at weaning increased by 10 cm, while daily weight gain increased by 2.1 kg/day. The height at weaning increased by 10 cm when the weaning weight increased by 2.6 kg/day.

### 3.4. Parasite Incidence

Prevalence of *Eimeria* spp. (coccidia) in the calves during the three different periods (30, 60 days, and at weaning); no significant difference was observed among dam lactation numbers (*p* > 0.05). However, the calves from LAC3 did not show infection with this parasite at 30 days of age (Table 5). Prevalence of *Eimeria* spp. in all the periods tested was not influenced by birth weight or colostrum quality (Gravity, density, IgG content [Colostrometer and Refractometer]) and composition (Protein, fat, NFS, and density) (*p* > 0.05).

### 3.5. Calves’ Clinical Treatments

The costs incurred during clinical treatments varied among treatments (*p* < 0.01). On average, the treatment cost was $7.5 (MX$127) per calf for calves from LAC1, $13.7 (MX$238) per calf for calves from LAC2, and $15.6 (MX$271) per calf for calves from LAC3.

The economic investment in the treatment was negatively related to the IgG concentration (Refractometer reading; *p* < 0.001), the °Brix percentage (*p* < 0.01), and tended to be negatively related to the Colostrometer reading (*p* = 0.06). The economic investment decreased by $5.6 (MX$98.0), while the IgG concentration (Refractometer reading) in colostrum increased by 20%. The economic investment decreased by $2.8 (MX$48.5), while the °Brix percentage increased by 2%. The economic investment decreased by $1.7 (MX$30.0), while the IgG concentration (Colostrometer reading) in colostrum increased by 20 mg/mL. The economic investment was not associated with colostrum composition (Protein, fat, NFS, and density) (*p* > 0.05).

### 3.6. Correlations

Correlation analysis demonstrated a structured relationship among colostrum quality indicators, chemical composition, growth performance, and economic investment (Table 6). Strong positive associations were observed among indirect measures of colostrum quality, particularly between °BRIX and refractometry (*r* = 0.964, *p* < 0.0001) and between °BRIX and gravity (*r* = 0.559, *p* < 0.0001). Colostrum density was strongly associated with protein concentration (*r* = 0.731, *p* < 0.0001) and non-fat solids (NFS; r = 0.550, *p* = 0.0001) and moderately associated with fat content (*r* = 0.347, *p* = 0.023). Protein concentration was also correlated with non-fat solids (*r* = 0.521, *p* = 0.0003) and fat content (*r* = 0.342, *p* = 0.025). In contrast, neonatal birth weight was not significantly associated with colostrum quality or compositional variables (*p* > 0.05). Postnatal performance was primarily characterized by a strong association between weaning weight and growth performance (*r* = 0.948, *p* < 0.0001), with additional moderate correlations with body height (weaning weight: *r* = 0.491, *p* < 0.001; gain weight: *r* = 0.403, *p* = 0.01). Economic investment showed significant negative correlations with major colostrum quality indicators, including °BRIX (*r* = −0.612, *p* < 0.0001) and refractometry (*r* = −0.561, *p* < 0.0001), as well as with body height (*r* = −0.369, *p* < 0.01).

## 4. Discussion

The present study evaluated the effects of dam lactation number on colostrum quality, calf growth performance, health status, and economic investment, providing a comprehensive assessment of perinatal development in dairy production systems. Overall, the results indicate that although lactation number did not markedly influence colostrum immunoglobulin (IgG) concentration, it significantly affected colostrum composition and calf growth performance, with important implications for herd productivity and management. Correlation analyses further revealed structured relationships among colostrum quality indicators, chemical composition, growth traits, and economic outcomes, suggesting strong methodological consistency and biological integration.

### 4.1. Colostrum Immunoglobulin Content and Chemical Composition

Contrary to expectations, colostrum IgG concentration, estimated using a colostrometer, refractometer, °Brix, and specific gravity, did not differ among lactation groups. These findings suggest that, under adequate nutritional and management conditions, cows produce colostrum of comparable immunological quality regardless of parity. Nevertheless, correlation analysis revealed a highly structured relationship among colostrum components, indicating that colostrum functions as an integrated system that reflects macronutrient composition and is coordinately regulated. Previous studies have reported variable effects of parity on colostrum IgG content, with multiparous cows often producing colostrum with higher immunoglobulin concentrations [9,10,34]. This may be attributed to the greater cumulative antigen exposure and enhanced mammary secretory capacity observed in multiparous cows compared with primiparous cows [9,10,11,12,13]. In this study, both primiparous and multiparous cows were provided the same diet ad libitum. Although individual feed intake was not measured, multiparous cows generally consume more feed than primiparous cows due to their larger body size and higher milk production potential [35]. Increased nutrient intake may enhance colostrum quality, as calostrogenesis is highly dependent on nutrient availability and is sensitive to maternal feed intake during late gestation [36]. Consequently, the greater voluntary feed intake of multiparous cows may have facilitated the synthesis and transfer of immune components into colostrum. Nevertheless, the absence of such differences in the present study may reflect uniform health status, optimized dry-period management, and effective prepartum nutrition across experimental groups [14,37,38]. These factors likely minimized variability in IgG concentration and immune transfer and contributed to the overall high colostrum quality observed. The strong correlation among multiple indirect methods for estimating IgG concentration reinforces the reliability of the measurements [10,26]. These techniques yield comparable estimates of colostrum quality and support their use as practical, reliable tools for assessing colostrum’s immunological value in research and at a farm level. The lack of treatment differences also suggests that passive immunity transfer was not a confounding factor in explaining subsequent differences in calf performance.

In contrast to immunoglobulin content, the chemical composition of colostrum was influenced by the number of lactations. Colostrum from LAC3 cows exhibited higher protein concentrations, whereas colostrum from LAC1 cows was characterized by elevated fat content. These findings are consistent with physiological adaptations associated with mammary gland maturity and metabolic status in multiparous cows [39]. Higher protein content in colostrum from older cows may reflect enhanced mammary secretory capacity and greater availability of circulating amino acids for milk synthesis [7,40,41]. The strong positive association between colostrum density and protein concentration supports the conclusion that protein is a primary determinant of colostrum density [42]. This observation is consistent with evidence indicating that diet quality, and consequently immunoglobulins and other proteins, constitute a substantial proportion of the solid fraction of colostrum [9]. These findings highlight the value of density-based measurements as practical indicators of colostrum quality and potential immunological efficacy. Conversely, the increased fat concentration observed in primiparous cows may be associated with altered energy partitioning and lipid metabolism during early lactation, as well as the dilutive effect previously reported across species [43,44,45]. This is supported by the relationship between density and fat content, which indicates that although lipids contribute to colostrum composition and energy value, their influence on density is less pronounced than that of the aqueous solid components [42]. Despite these compositional differences, density and NFS were similar among treatments, indicating that overall colostrum solids remained relatively stable. This suggests that variations in protein and fat were balanced within the broader matrix of colostrum constituents, maintaining structural and nutritional consistency [7]. Importantly, these compositional changes did not translate into differences in the transfer of passive immunity, reinforcing the notion that immunological quality is not solely determined by gross nutrient composition.

A limitation of the current experiment is the lack of data on colostrum yield. Although all the cows calved within the same month, allowing us to avoid seasonal effects on colostrum composition [13], the colostrum yield was not recorded in the present study. The primary objective of this study was to evaluate changes in colostrum quality rather than quantity, as we knew that the number of lactations would influence colostrum yield [13,30].

### 4.2. Birth Weight and Postnatal Growth Performance

Calf birth weight and growth performance were strongly influenced by the maternal lactation group. Calves born to LAC1 dams exhibited lower birth weight, reduced weight gain, lower weaning weight, and diminished skeletal growth compared with calves from LAC2 and LAC3 dams. Lower birth weight in calves from primiparous cows may be attributed to reduced uterine capacity, limited placental development, and greater competition for nutrients between maternal growth and fetal demands [46,47]. These constraints can result in an inadequate nutrient supply to the developing fetus, potentially causing long-lasting effects on postnatal growth and performance. Our findings reinforce our previous findings and those of others, indicating that maternal maturity and physiological status exert substantial effects on fetal development and postnatal growth [10,48,49,50]. The observed positive association between birth weight and weaning weight supports the notion that prenatal advantages can persist into early growth stages [51,52]. This association is further substantiated by positive correlations among weaning weight, weight gain, and body height, indicating that animals with greater body mass at weaning also demonstrate enhanced structural development and growth rates. These findings align with previous studies showing that faster-growing animals are typically both heavier and taller at weaning, which underscores the interdependence of body weight gain and skeletal growth during early development [53,54]. These results highlight the importance of optimal fetal development in shaping growth trajectories that influence subsequent productive performance.

An additional consideration is sire selection. In dairy systems, primiparous cows are often bred to sires with lower expected birth weights to reduce the risk of dystocia [55,56]. Although only Holstein semen was used in this study, sires selected for calving ease may have contributed to the lower birth weights observed in LAC1 calves. Nevertheless, the overall pattern remains consistent with established literature indicating lighter calves from first-parity dams [49,50]. Interestingly, preweaning growth and weaning weight were not directly influenced by colostrum quality or composition, confirming previous reports [57]. Growth performance in pre-weaned dairy calves is influenced by several factors, such as the quantity and quality of the liquid diet, early solid feed intake, and genetic potential [58]. Providing sufficient nutritional support during the neonatal period is essential for optimal growth and development, whereas nutritional deficiencies can negatively affect health, growth performance, and survival [5]. Furthermore, skeletal development, as reflected by weaning height, which is an important tool in the dairy industry, was positively associated with colostrum IgG concentration. This relationship suggests that passive immunity may indirectly support structural growth by reducing early-life disease burden and improving nutrient utilization [20,59,60]. Enhanced immune protection likely allows calves to allocate more energy toward growth rather than immune responses. The observed linear relationships between IgG concentration, weight gain, weaning weight, and skeletal growth further emphasize the biological relevance of early immune status for long-term developmental outcomes. Nevertheless, more research is needed to elucidate the relationship between IgG concentration and weaning height and at first service.

### 4.3. Eimeria spp. Prevalence and Immune Protection

The prevalence of *Eimeria* spp. did not differ significantly among treatments or sampling periods, indicating comparable exposure and infection risk across groups. The absence of infection in LAC3 calves at 30 days suggests a transient protective effect, potentially linked to improved maternal immunity, early-life resilience, or management-related factors [61]. Nevertheless, the lack of association between colostrum quality and *Eimeria* spp. prevalence supports previous observations that coccidiosis is primarily influenced by environmental contamination, housing hygiene, and stocking density rather than passive immunity alone [61]. Because *Eimeria* spp. are intracellular parasites that elicit primarily cell-mediated immune responses, humoral immunity transferred via colostrum may play a limited role in conferring protection [62,63]. These findings underscore the importance of integrating colostrum management with environmental control strategies to mitigate enteric parasite infections in young calves.

### 4.4. Economic Investment and Production Relevance

Treatment costs differed significantly among lactation groups, with calves from LAC3 dams incurring the highest medical expenses. Although these calves exhibited superior growth performance, increased health interventions may reflect greater metabolic demands or management-related factors associated with higher-producing cows. Notably, treatment costs were inversely related to colostrum IgG concentration and °Brix percentage. This was confirmed by the negative correlations between economic investment and colostrum quality indicators, as well as with body height. Improved colostrum quality was associated with substantial reductions in veterinary expenses, highlighting the economic value of effective passive immunity transfer. These findings are consistent with previous reports demonstrating that adequate colostrum management reduces morbidity, mortality, and long-term production losses [7,64]. The observed reductions in treatment costs with increasing IgG and °Brix percentage provide quantifiable evidence of the financial benefits of monitoring and optimizing colostrum quality at the farm level. Simple, low-cost tools such as refractometers and colostometers therefore represent valuable management tools for improving herd profitability.

### 4.5. Implications for Dairy Management and Future Research

Collectively, the results indicate that lactation number influences colostrum composition and calf growth performance but does not necessarily compromise immunological quality. While primiparous cows produced colostrum with adequate IgG levels, their calves exhibited inferior growth, reinforcing the relationship between maternal body weight and neonatal birth weight. This emphasizes the need for targeted nutritional and management strategies for young dams. The positive associations between IgG concentration, skeletal development, and reduced treatment costs highlight the multifaceted role of colostrum in shaping biological and economic outcomes. These findings highlight the need to adapt and implement early colostrum assessment and quality control at the farm. Additionally, further monitoring protocols to assess growth rates and for infectious diseases and diarrhea are necessary. Future research should explore the mechanistic links between passive immunity, endocrine regulation, and tissue accretion, as well as the long-term effects of early-life immune status on lifetime productivity. Additionally, integrating genomic, nutritional, and microbiome data may further clarify maternal-offspring interactions in dairy systems.

## 5. Conclusions

In conclusion, this study demonstrates that although lactation number does not significantly affect colostrum IgG concentration, it influences colostrum nutrient composition and calf growth trajectories. The cow’s parity affected the quality of colostrum produced, particularly the protein and fat content. Calves from multiparous cows exhibited superior prenatal and postnatal development, whereas higher colostrum IgG levels were associated with improved skeletal growth and reduced health-related costs. These findings emphasize the central role of colostrum management and further monitoring protocols in optimizing calf performance and economic sustainability in dairy production systems.

## Figures and Tables

**Table 1:** Ingredients in the diet provided to primiparous and multiparous cows during the experiment.

Ingredient	Primiparous (Kg)	Primiparous (%)	Multiparous (Kg)	Multiparous (%)
Rolled corn	1.39	4.5	1.75	6.4
Soybean	1.30	4.2	1.60	5.9
Canola	1.20	3.9	0.50	1.8
Minerals	0.25	0.8	0.80	2.9
Corn silo	24.00	77.1	20.00	73.7
Hay	3.0	9.6	2.50	9.2
Total	31.14	100	27.15	100

**Table 2:** The effect of dam lactation numbers (First lactation: LAC1; second lactation: LAC2; third lactation: LAC3) on colostrum IgG concentration (means ± SEM) assessed by colostrometer and refractometer, °Brix percentage in colostrum, and colostrum gravity.

Variable		LAC1	LAC2	LAC3	SEM	*p*-Value
	n	15	15	15		
Gravity		1060	1056	1062	1.54	0.28
°Brix percentage		27.5	25.3	27.7	0.547	0.12
Colostrometer IgG (mg/mL)		86.3	76.1	91.4	3.93	0.28
Refractometer IgG (g/L)		92.2	81.3	88.1	2.45	0.18

**Table 3:** The effect of dam lactation numbers (First lactation: LAC1; second lactation: LAC2; third lactation: LAC3) on colostrum chemical composition (Protein, Fat, NFS, Density [means ± SEM]).

Variable		LAC1	LAC2	LAC3	SEM	*p*-Value
	n	15	15	15		
Protein (%)		4.8 ^a^	5.4 ^ab^	6.8 ^b^	0.31	0.02
Fat (%)		5.7 ^a^	3.4 ^ab^	2.9 ^b^	0.46	0.03
NFS (%)		13.7	22.2	22.6	1.66	0.23
Density (g/mL)		48.5	52.7	53.7	2.93	0.76

NFS: Non-Fat Solids. ^a,ab,b^ Values within the same variable and period with a different superscript letter differ (*p* < 0.05).

**Table 4:** The effect of dam lactation numbers (First lactation: LAC1; second lactation: LAC2; third lactation: LAC3) on birth weight (BWT), weaning weight (WWT), weight gain (WTG), and height. Values are the mean. SEM: Standard Error of the Mean.

Variable		LAC1	LAC2	LAC3	SEM	*p*-Value
	n	15	15	15		
BWT (Kg)		30.8 ^a^	38.5 ^b^	41.3 ^c^	0.83	0.0001
WWT (Kg)		71.2 ^a^	85.6 ^b^	95.6 ^c^	2.11	0.0001
WTG (kg)		40.4 ^a^	47.1 ^ab^	54.4 ^b^	1.72	0.002
Height (cm)		87.3 ^a^	91.5 ^b^	96.5 ^c^	0.97	0.002

*^a,ab,b,c^ Values within the same variable and period with a different superscript letter differ (p < 0.05 to 0.001).*

**Table 5:** The effect of dam lactation numbers (First lactation: LAC1; second lactation: LAC2; third lactation: LAC3) on the prevalence of *Eimeria* spp. (coccidia) in the calves.

Variable		LAC1	LAC2	LAC3	*p*-Value
	n	15	15	15	
Prevalence at 30 days (%)		27	40	0	0.74
Prevalence at 60 days (%)		13	47	27	0.16
Prevalence at 90 days (%)		33	47	20	0.32

**Table 6:** Correlations (*r*; *p*-value) among colostrum gravity (Gravity), colostrum Ig ^1^ concentration (colostrometer), °Brix percentage (refractometer), colostrum Ig ^2^ concentration (refractometer), colostrum density (Den), colostrum protein (protein), colostrum fat (Fat), colostrum non-fat solids (NFS), birth weight (BWT), weaning weight (WWT), weight total gain (WTG), height at weaning (Height), and economic investment ($$) from dams with different lactation numbers (First lactation: LAC1; second lactation: LAC2; third lactation: LAC3). The data combined all the dams with different lactation numbers.

	Gravity	IG ^1^	°Brix	IG ^2^	Den	Protein	Fat	NFS	BWT	WWT	WTG	Height	$$
Gravity		1.0 (<0.001)	0.55 (<0.001)	0.49 (<0.01)	0.13 (0.42)	−0.5 (0.72)	−0.20 (0.19)	0.12 (41)	0.13 (38)	0.15 (32)	0.11 (0.48)	0.38 (0.01)	−0.28 (0.06)
IG ^1^			0.55 (<0.001)	0.49 (<0.01)	0.13 (0.42)	−0.5 (0.72)	−0.20 (0.19)	0.12 (41)	0.13 (38)	0.15 (32)	0.11 (0.48)	0.38 (0.01)	−0.28 (0.06)
°Brix				0.96 (<0.001)	0.16 (0.28)	−0.02 (0.89)	−0.23 (0.13)	0.04 (0.77)	−0.06 (0.70)	0.05 (0.73)	0.07 (0.65)	0.33 (0.02)	0.55 ()
IG ^2^					0.17 (0.25)	0.01 (0.92)	−0.15 (0.32)	0.06 (0.67)	−0.08 (0.58)	−0.01 (0.99)	0.02 (0.87)	0.32 (0.03)	0.56 (<0.001)
Den						0.73 (<0.001)	0.35 (0.02)	0.55 (<0.001)	−0.07 (0.63)	−0.09 (0.54)	−0.07 (0.65)	0.01 (0.98)	−0.15 (0.32)
Protein							0.34 (0.02)	0.52 (< 0.01)	0.03 (0.82)	−0.24 (0.11)	0.25 (0.10)	−0.11 (0.48)	0.13 (0.41)
Fat								0.16 (0.30)	0.14 (0.35)	0.02 (0.85)	−0.01 (0.91)	−0.15 (0.34)	0.19 (0.20)
NFS									0.06 (0.70)	−0.22 (0.15)	−0.24 (0.12)	0.02 (0.87)	0.15 (0.31)
BWT										0.12 (0.42)	−0.19 (0.20)	0.25 (0.10)	0.06 (0.66)
WWT											0.94 (<0.001)	0.49 (<0.01)	0.01 (0.97)
WTG												0.40 (<0.01)	−0.01 (0.91)
Height													−0.36 (0.01)
$$													

## Data Availability

The data presented in the manuscript were part of Andrea García-Mendoza’s Thesis Project. The raw data supporting the conclusions of this article will be made available by the authors upon request.
